# Nutrient deprivation alters the rate of COPII coat assembly to tune secretory protein transport

**DOI:** 10.21203/rs.3.rs-2652351/v1

**Published:** 2023-03-17

**Authors:** William Kasberg, Peter Luong, Kevin A. Swift, Anjon Audhya

**Affiliations:** 1 Department of Biomolecular Chemistry, University of Wisconsin School of Medicine and Public Health, Madison, WI, 53706, USA

**Keywords:** protein secretion, membrane trafficking, Sec23, TFG, lattice light-sheet microscopy

## Abstract

Co-assembly of the multilayered coat protein complex II (COPII) with the Sari GTPase at subdomains of the endoplasmic reticulum (ER) enables secretory cargoes to be concentrated efficiently within nascent transport intermediates, which subsequently deliver their contents to ER-Golgi intermediate compartments. Here, we define the spatiotemporal accumulation of native COPII subunits and secretory cargoes at ER subdomains under differing nutrient availability conditions using a combination of CRISPR/Cas9-mediated genome editing and live cell imaging. Our findings demonstrate that the rate of inner COPII coat assembly serves as a determinant for the pace of cargo export, irrespective of COPII subunit expression levels. Moreover, increasing inner COPII coat assembly kinetics is sufficient to rescue cargo trafficking deficits caused by acute nutrient limitation in a manner dependent on Sar1 GTPase activity. Our findings are consistent with a model in which the rate of inner COPII coat formation acts as an important control point to regulate cargo export from the ER.

## Introduction

The vast majority of newly synthesized secretory cargoes are translated into the lumen of the endoplasmic reticulum (ER) and recognized by a variety of receptors that promote their packaging into transport intermediates (1–3). The prototypical cargo receptor Sec24 functions as part of a multi-subunit complex of coat proteins collectively known as COPII, which assembles immediately adjacent to ER subdomains harboring the scaffolding protein Sec16a, members of the Tango1/cTAGE family, and the guanine nucleotide exchange factor Sec12 (3–16). Based on numerous studies over the past several decades, a detailed model has evolved to describe the earliest steps of COPII-mediated trafficking. Specifically, Sec12 first promotes GTP loading onto the small GTPase Sar1, enabling stable penetration of its amino-terminal amphipathic helix into the lipid bilayer to induce membrane tubulation while simultaneously directing the recruitment and organization of Sec23-Sec24 heterodimers to form the inner layer of the COPII coat complex. (6, 17–25). Both Sec16a and Tango1/cTAGE also interact directly with Sec23-Sec24 and have been suggested to increase their local concentration, thereby stimulating coat formation (14, 26, 27). Sec16a additionally associates with Sec13-Sec31 heterotetramers, which co-assemble around the inner coat to form an outer COPII cage (28, 29). This dual-layered coat complex has been proposed to sculpt membrane tubules generated by activated Sar1 to form nascent cargo-laden transport intermediates (29–36). In a manner that is likely dependent on Sar1 GTP hydrolysis, these intermediates undergo maturation and subsequently deliver their contents to ER-Golgi intermediate compartments (ERGIC), which is facilitated by the Sec23-binding protein TFG (37–43).

The Sar1 GTPase cycle has also been implicated in controlling the timing of COPII coat disassembly, which is required prior to transport intermediate fusion with ERGIC membranes (3, 44, 45). Somewhat paradoxically, the inner coat protein Sec23 functions as the guanine nucleotide activating protein (GAP) for Sar1, inserting an arginine finger into its active site, which is further stimulated by the outer coat subunit Sec31 (30, 33, 45–48). Therefore, regulatory mechanisms must exist to tightly control Sec23 GAP activity during transport intermediate formation, both to prevent premature COPII coat disassembly, but also to maintain a rapid rate of anterograde cargo transport at the ER/ERGIC interface. Several cell signaling pathways have been suggested to function in this context. Governed by nutrient availability, the presence of growth factors, cellular metabolism, and various forms of cell stress, COPII subunits and multiple COPII regulatory factors undergo post-translational modifications, including phosphorylation, glycosylation, and ubiquitylation, leading to alterations in their local concentrations at sites of transport intermediate formation, which in turn tunes the kinetics of cargo export (49–60). In particular, acute serum and amino acid deprivation have been demonstrated to alter the phosphorylation state of several COPII subunits as well as Sec16a, reducing their levels at ER subdomains and impairing the rate of COPII-mediated cargo transport (49, 50). Similarly, reduced activity of the IRE1 branch of the unfolded protein response (UPR) pathway resulting from prolonged absence of nutrients was shown to downregulate the expression of numerous factors involved in COPII-mediated trafficking, including Sec16a and isoforms of Sec23, Sec24, and Sec31 (61).

Despite our expansive understanding of the regulatory systems that control post-translational modifications on COPII subunits and their levels of expression under various conditions, the dynamics of COPII assembly and disassembly remain poorly defined. This is in part due to the relative dearth of live cell imaging studies examining native COPII subunits. Instead, the majority of published work has focused on fixed cell analysis or the use of ectopic overexpression, which can alter protein dynamics, to determine COPII distribution. Here, we leverage CRISPR/Cas9-mediated genome editing to establish a series of human cell lines that endogenously express HaloTag fusions to several COPII subunits, as well as key COPII regulatory factors. In combination with lattice light-sheet imaging, we define the rates of COPII subunit incorporation at ER subdomains and demonstrate that acute nutrient deprivation slows the kinetics of Sec23 addition. This defect is accompanied by a diminished rate of anterograde cargo transport, consistent with previous work (49, 52, 61). However, by artificially increasing the rate of Sec23 incorporation, we show that cargo trafficking defects induced by short-term nutrient deprivation can be rescued in a manner that requires its GAP activity. Taken together, our findings are most consistent with a model in which the rate of inner COPII coat assembly dictates the kinetics of secretory cargo packaging and export from the ER.

## Results

### COPII subunits endogenously appended with HaloTag function normally in secretory protein trafficking

**Figure 1.**
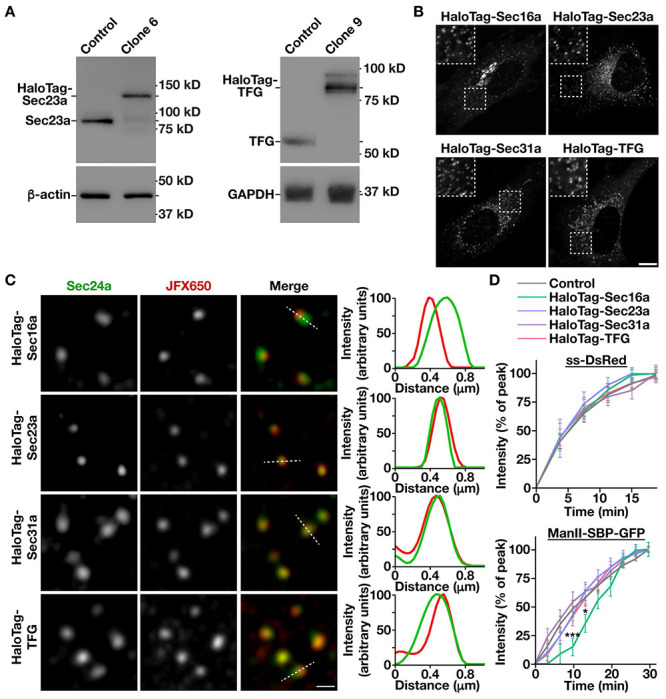
Engineering human cell lines to study native COPII dynamics. (A) Representative immunoblots (n=3) of extracts generated from control and clonal CRISPR/Cas9 edited cell lines using antibodies directed against Sec23a (left, top), β-actin (left, bottom), TFG (right, top) and GAPDH (right, bottom). (B) Representative confocal images of cell lines natively expressing HaloTag fusion proteins after labeling with JFX650-HaloTag ligand are shown. Insets show 3x zoomed regions (boxed). Bar, 10 μm. (C) Representative super resolution images of fixed cells expressing HaloTag fusion proteins labeled with the JFX650-HaloTag ligand and co-stained using antibodies directed against Sec24a. Linescans highlighting their relative localizations are shown (right). Bar, 500 nm. (D) Confocal imaging of edited cell lines co-expressing native HaloTag fusion proteins and either ss-DsRed (top) or ManII-SBP-GFP (bottom) was used to monitor their synchronous release from the ER. Based on fluorescence intensity, the percentage of each cargo present within the perinuclear region relative to its maximal accumulation there was quantified over time. Error bars represent mean +/− SEM (n=20 cells each; 3 biological replicates each). ***, p < 0.001 and *, p < 0.05, as calculated using a two-way ANOVA and Tukey post hoc test.

Although COPII-mediated trafficking has been successfully reconstituted in vitro (62), relatively little is known about the regulatory mechanisms that govern the rate of cargo egress from the ER in mammalian cells. To address this issue, we first leveraged CRISPR/Cas9 genome editing to append the modular HaloTag on several factors implicated directly in regulating the anterograde transport of newly synthesized secretory proteins. In particular, we focused on the scaffolding protein Sec16a, which marks subdomains of the ER from which secretory cargoes emerge, components of the inner and outer COPII coat, Sec23a and Sec31a respectively, and the Sec23-binding protein TFG, which has been suggested to organize COPII transport intermediates at the ER/ERGIC interface (Supplementary Fig. 1a). HaloTag was selected due to its unique ability to rapidly and irreversibly bind to cell permeable ligands that are coupled to bright and photostable fluorescent dyes, making them ideal for live cell imaging (63). CRISPR/Cas9 editing was conducted using immortalized human Retinal Pigment Epithelial (RPE1) cells, which exhibit a stable diploid karyotype, and multiple clones expressing each fusion protein in the absence of overexpression were identified using immunoblot analysis and sequencing. Individual clones expressing native levels of HaloTag-Sec23a, HaloTag-Sec31a, and HaloTag-TFG in a homozygous manner were selected for further study, as was a clone expressing HaloTag-Sec16a from a single allele, since we were unable to identify any that were edited homozygously (Fig. 1a and Supplementary Fig. 1b, c).

To verify the functionality of each fusion protein, we first examined the proliferation rates of the edited cell lines, since ongoing COPII-mediated trafficking is essential for viability (3). With the exception of cells expressing HaloTag-Sec16a, which grew significantly slower, all other cell lines exhibited a doubling time that was similar to control, unedited RPE1 cells (Supplementary Fig. 1d). We next determined the distributions of each fusion protein using confocal and super-resolution fluorescence microscopy, which showed that they all localized appropriately at or juxtaposed to sites decorated with antibodies directed against endogenous Sec24a, another component of the COPII coat (Fig. 1b, c and Supplementary Fig. 1e). Linescan analysis further confirmed these findings, strongly suggesting that the addition of HaloTag does not interfere with targeting of the fusion proteins (Fig. 1c). Importantly, organization of the early secretory pathway was not impacted by their expression, as indicated by normal distributions of ER, ERGIC, and Golgi markers, as compared to control RPE1 cells (Supplementary Fig. 2).

To assess whether the rate of anterograde trafficking was affected in cells expressing HaloTag fusion proteins, we conducted two distinct synchronized cargo release assays (64, 65). In the first case, an ER export sorting motif appended onto DsRed (ss-DsRed) was expressed as a fusion to a mutant form of the FK506-binding protein (FKBP) that causes its aggregation in the ER lumen. Only upon solubilization mediated by the addition of synthetic ligand of FKBP (SLF) does ss-DsRed undergo packaging into COPII transport intermediates for export from the ER (64). In a second approach, the human invariant chain of the major histocompatibility complex fused to streptavidin, which is translated into the ER lumen but unable to leave, was co-expressed with a fragment of the Golgi resident enzyme Mannosidase II fused to streptavidin binding protein and GFP (ManII-SBP-GFP), restricting it to the ER lumen until the addition of biotin enables dissociation and subsequent export (65). Live cell confocal imaging was used to monitor movement of ss-DsRed and ManII-SBP-GFP following release in control and CRISPR/Cas9-edited cell lines. We found that neither cargo was affected by the expression of HaloTag fusion proteins, with the exception of a brief delay in ManII-SBP-GFP trafficking in cells expressing HaloTag-Sec16a (Fig. 1d and Supplementary Fig. 3). Taken together, our findings strongly suggest that Sec23a, Sec31a, and TFG retain functionality when appended with the HaloTag, while Sec16a function may be partially perturbed under this condition.

### Components involved in COPII-mediated trafficking exhibit distinct assembly kinetics at ER subdomains

**Figure 2.**
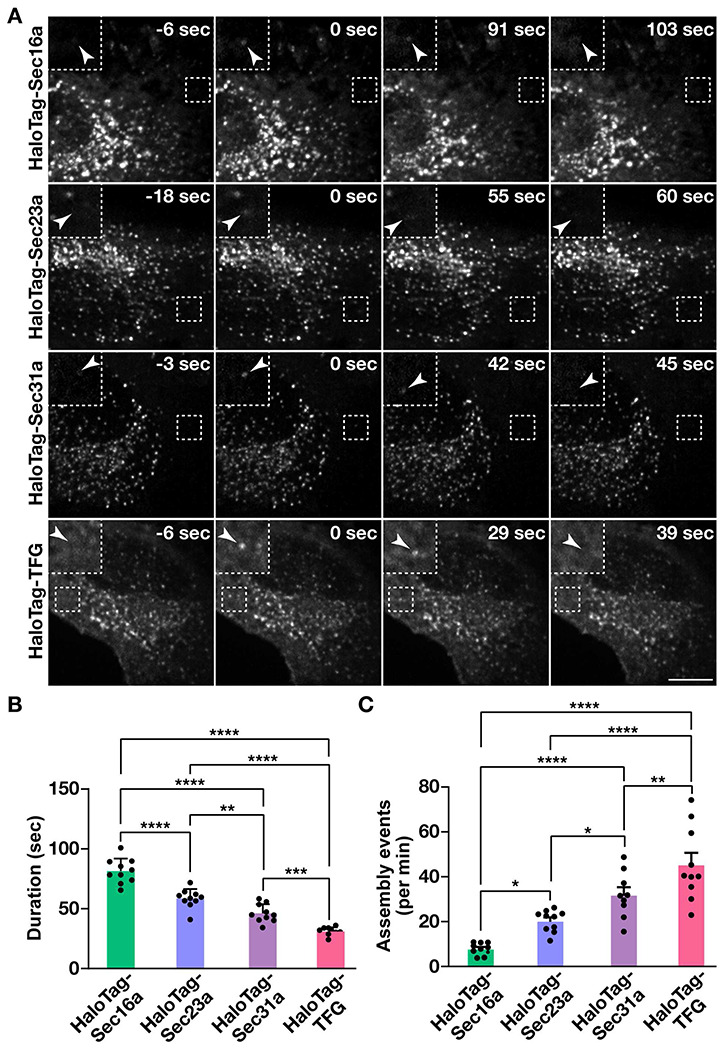
COPII dynamics under nutrient replete conditions. (A) Representative timelapse series showing the distributions of HaloTag fusion proteins following labeling with the JFX650- HaloTag ligand. Insets (3x zoom) highlight structures indicated by arrowheads that assemble and disassemble during the imaging window. Bar, 10 μm. (B and C) Quantification of the average duration of each HaloTag fusion protein at an ER subdomain (B) and the number of structures that assemble each minute (C). Error bars represent mean +/− SEM (n=10 cells each; 3 biological replicates; more than 3000 tracked particles each). ****, p < 0.0001; ***, p < 0.001; **, p < 0.01 and *, p < 0.05, as calculated using a one-way ANOVA and Tukey post hoc test.

The establishment of cell lines natively expressing tagged isoforms of key regulators that direct anterograde secretory protein transport affords the unique opportunity to define the manner in which these factors accumulate at ER subdomains to influence cargo export. For these studies, we leveraged lattice light-sheet microscopy, an approach that provides near diffraction-limited resolution and collects full cell volumes with high speed and low levels of phototoxicity as compared to other forms of live cell imaging. Each cell line was labeled using fluorogenic HaloTag ligands and imaged continuously for 10 minutes. Consistent with previous findings (66, 67), we identified both long-lived and short-lived sites harboring each marker (Supplementary Movies 1–4). To simplify analysis, we focused specifically on those structures that assembled and disassembled during the imaging window (Fig. 2a).

Using Imaris software, we were then able to measure the lengths of their individual lifetimes, which indicated that Sec16a exhibits significantly more stability at ER subdomains as compared to either COPII subunit or TFG (Fig. 2b). Additionally, we found that the inner COPII coat persists for a longer period of time than the outer coat, while TFG acts most transiently during cycles of COPII assembly and disassembly (Fig. 2b). We also measured how often new sites appeared during the imaging period and found that TFG-positive structures, while shortest lived at ER subdomains, exhibited the highest frequency of de novo formation (Fig. 2c). In contrast, assembly of new Sec16a-labeled sites was relatively infrequent (Fig. 2c).

**Figure 3.**
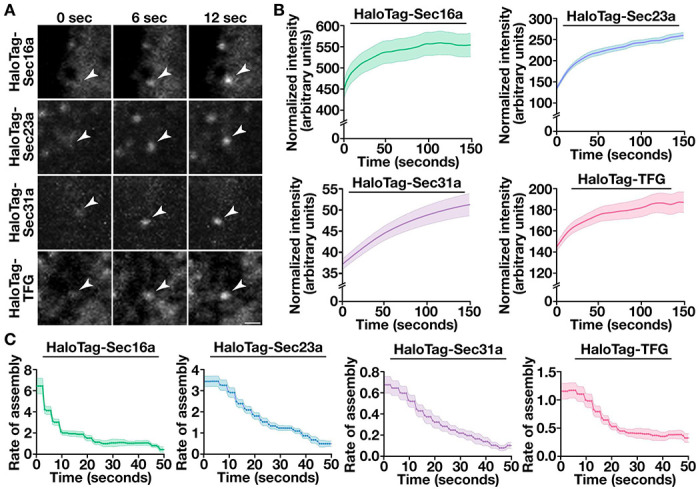
Assembly kinetics of COPII components and associated regulatory factors. (A) Representative timelapse series using lattice light-sheet imaging showing the assembly of HaloTag fusion proteins following labeling with the JFX650-HaloTag ligand. Arrowheads highlight structures undergoing assembly in cells. Bar, 1 μm. (B and C) Quantification of the normalized intensity (B) and instantaneous rate of assembly (C) of tracked HaloTag fusion proteins. Error, as displayed by lightly colored bands around smoothed tread lines, represents mean +/− SEM (n=10 cells each; 3 biological replicates; more than 3000 tracked particles each).

We next quantified the intensities of individual structures as they formed, yielding an assembly curve for the HaloTag fusion proteins (Fig. 3a and Supplementary Fig. 4). Each had shared characteristics, including an early period of increasing intensity, which was followed by a plateau phase. As the number of sites that could be analyzed diminished due to disassembly, the plateau phases became more stochastic with time (Supplementary Fig. 4). We therefore focused specifically of their assembly and calculated their instantaneous rates of change during the first 50 seconds of formation (Fig. 3b, c). These data demonstrated that Sec16a assembles most rapidly, but transitions quickly toward a slower rate of subunit incorporation. Moreover, Sec23a and Sec31a exhibited highly distinct rates of assembly, with Sec31a subunit addition being much slower than that of the inner coat (Fig. 3c). Together, these data show that each regulator of COPII-mediated trafficking has a distinctive lifetime, frequency of formation, and rate of incorporation, and that these kinetics can be defined using high spatiotemporal imaging.

### Nutrient availability influences the kinetics of COPII assembly

**Figure 4.**
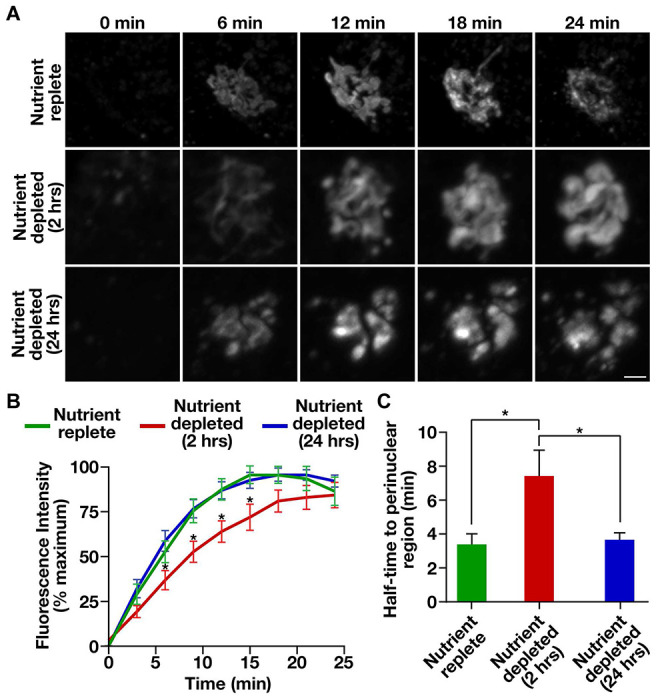
Nutrient deprivation influences the rate of cargo egress from the ER. (A) Control cells expressing ss-DsRed were maintained under nutrient replete conditions or subjected to acute (2 hours) or long-term (24 hours) nutrient deprivation prior to SLF treatment and live cell timelapse imaging. Representative images zoomed to the perinuclear region of cells are shown at various timepoints. Bar, 1 μm. (B) Confocal imaging of control cells expressing ss-DsRed under various conditions was used to monitor its synchronous release from the ER. Based on fluorescence intensity, the percentage of ss-DsRed present within the perinuclear region relative to its maximal accumulation there was quantified over time. Error bars represent mean +/− SEM (n=20 cells each; 3 biological replicates each). *, p < 0.05, as calculated using a two-way ANOVA and Tukey post hoc test. (C) Quantification of the half-time to perinuclear accumulation of ss-DsRed under various conditions. Error bars represent mean +/− SEM (n=20 cells each; 3 biological replicates each). *, p < 0.05, as calculated using a one-way ANOVA and Tukey post hoc test.

Previous studies have shown that acute nutrient limitation reduces the rate of secretory cargo trafficking from the ER, with some ascribing this effect to changes in the expression levels of key COPII regulatory factors and/or their post-translational modification (49, 50, 61). Using ss-DsRed and ManII-SBP-GFP as model secretory cargoes, we confirmed that RPE1 cells deprived of nutrients for two hours exhibited a delay in transport to the perinuclear Golgi (Fig. 4, Supplementary Fig. 5a–c, and Supplementary Movies 5 and 6). Surprisingly however, cells that underwent prolonged nutrient deprivation failed to show a similar effect on the trafficking of ss-DsRed. Instead, the rate of ss-DsRed transport to the perinuclear region of cells grown in the absence of nutrients for 24 hours was comparable to that found in control cells maintained under nutrient replete conditions (Fig. 4, Supplementary Fig. 5a–c, and Supplementary Movies 5 and 7). Similarly, another cargo capable of induced release from the ER (HaloTag-L1CAM) and exhibited slowed transport following 2 hours of nutrient depletion relative to nutrient replete conditions, recovered in transport rate after 24 hours of nutrient deprivation (Supplementary Fig. 5d, e). One notable difference between control and nutrient deprived cells was the morphology of cargoes that accumulated near the perinuclear region of cells, which exhibited a fragmented appearance when nutrients were limiting (Fig. 4a). To determine whether this phenotype could be attributed to a change in Golgi morphology under these conditions, we used CRISPR/Cas9 editing to append a HaloTag onto endogenous GRASP65, a Golgi matrix protein involved in cisternal stacking, and performed super-resolution imaging in the presence and absence of nutrients. Our findings revealed that the highly interconnected, ribbon-like morphology of the Golgi was dramatically altered following both 2 and 24 hours of nutrient deprivation (Supplementary Fig. 5f), clarifying why the morphology of cargoes near the perinuclear region was perturbed under nutrient limiting conditions.

Our finding that extending the duration of nutrient deprivation leads to remediation of a cargo trafficking deficit is inconsistent with current models. Previous work showed that glucose starvation leads to downregulation of XBP1 splicing, which normally promotes the expression of several factors involved in COPII-mediated trafficking, including Sec16a and multiple COPII coat subunits (61). To determine whether prolonged nutrient deprivation may alter levels of spliced XBP1 (sXBP1), we conducted quantitative PCR studies. However, we found no significant change in sXBP1 mRNA levels in cells lacking nutrients for 2 or 24 hours as compared to controls (Supplementary Fig. 6a). Consistent with this finding, expression of another effector of sXBP1, the ER chaperone GRP78/BiP, was unchanged between 2 and 24 hours of nutrient deprivation (Supplementary Fig. 6b). We next directly examined expression of COPII in the presence and absence of nutrients, using immunoblotting analysis and quantitative live cell imaging. Consistent with prior work (61), the total cellular levels of both inner and outer COPII subunits were downregulated following 2 hours of nutrient deprivation and, in the case of the inner coat, were reduced further following 24 hours under these conditions (Supplementary Fig. 6c–e). In contrast, TFG levels were not affected by a short-term decrease in nutrient availability, but they were significantly reduced after prolonged absence of nutrients (Supplementary Fig. 6c–e). Together, these data argue against the idea that the overall expression levels COPII coat components or their regulators directly controls the rate of secretory cargo export.

To determine whether short- and/or long-term nutrient deprivation may have specific impacts on COPII at individual ER subdomains, we conducted a series of quantitative, live-cell imaging studies using confocal microscopy. We found that the total number of sites decorated with COPII subunits was reduced following prolonged nutrient deprivation, but their intensities were significantly elevated as compared to control cells and cells subjected to a short-term loss of nutrients (Supplementary Fig. 7). Similarly, the levels of Sec16a and TFG at ER subdomains were significantly elevated with long-term nutrient deprivation (Supplementary Fig. 7). These data raise the possibility that the local concentration of COPII and/or its regulators at ER subdomains may serve as a key determinant that governs the rate of cargo export.

**Figure 5.**
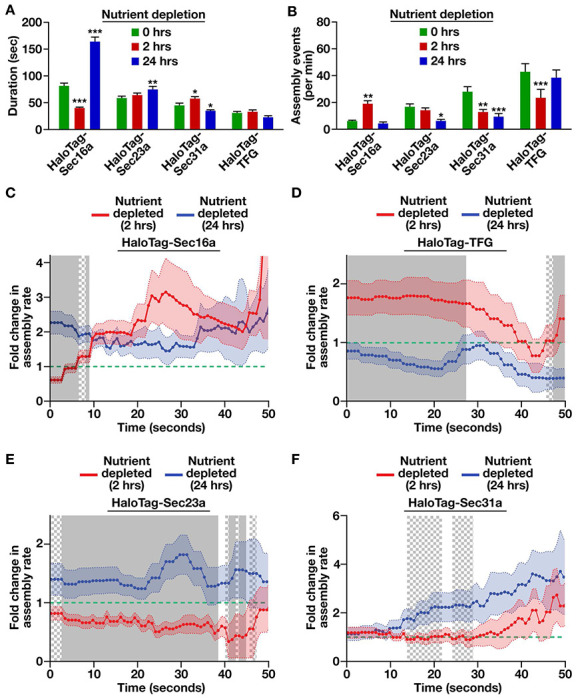
Short-term nutrient deprivation slows inner COPII coat assembly. (A and B) Quantification of the average duration of each HaloTag fusion protein at an ER subdomain under various nutrient availability conditions (A) and the number of structures that assemble each minute under those conditions (B). Error bars represent mean +/− SEM (n=10 cells each; 3 biological replicates; more than 10,000 tracked particles each). ****, p < 0.0001; ***, p < 0.001; **, p < 0.01 and *, p < 0.05, as calculated using a one-way ANOVA and Tukey post hoc test. (C-F) Quantification of the fold change in HaloTag fusion protein assembly rates following 2 and 24 hour nutrient deprivation relative to nutrient replete conditions (represented by a dashed green line). Error, as displayed by lightly colored bands around smoothed tread lines, represents mean +/− SEM (n=10 cells each; 3 biological replicates; more than 10,000 tracked particles each). Solid gray (p < 0.05) and checkered gray (p < 0.1) regions represent significant differences, calculated using multiple, unpaired *t* tests at each time point.

To investigate this idea, we measured the assembly kinetics of each HaloTag fusion protein under differing nutrient availability conditions. Following two hours of nutrient deprivation, the lifetime of Sec16a at ER subdomains decreased significantly as compared to control cells, although its frequency of formation increased, suggesting a high rate of turnover under this condition (Fig. 5a, b). In contrast, after 24 hours of nutrient deprivation, Sec16a-labeled sites became hyperstabilized, with a significantly extended lifespan as compared to control cells (Fig. 5a). Similarly, the lifetime of Sec23a-positive structures increased at the 24 hour timepoint, although their frequency of formation declined (Fig. 5a, b). Moreover, the outer COPII coat exhibited a diminished lifetime and frequency of formation following 24 hours of nutrient deprivation, despite the rate of cargo trafficking returning to that of nutrient replete conditions (Fig. 5a, b).

We next conducted an analysis of individual assembly curves, finding that the rate of Sec16a incorporation at ER subdomains was surprisingly elevated following both short- and long-term nutrient deprivation as compared to control cells (Fig. 5c). In contrast, the rate of TFG addition was significantly higher during short-term nutrient elimination as compared to control cells or cells subjected to a long-term absence of nutrients (Fig. 5d). Perhaps most strikingly, Sec23a incorporation at ER subdomains was dramatically enhanced following 24 hours of nutrient deprivation, as compared to control cells or cells depleted of nutrients for 2 hours (Fig. 5e), with Sec31a exhibiting a similar, albeit more modest, trend (Fig. 5f). These data suggest that the rate of Sec23a assembly at ER subdomains may serve as a key control point in determining the efficiency of anterograde transport of newly synthesized secretory cargoes.

### Artificially increasing the rate of Sec23a assembly bypasses cargo trafficking deficits that occur following short-term nutrient deprivation

**Figure 6.**
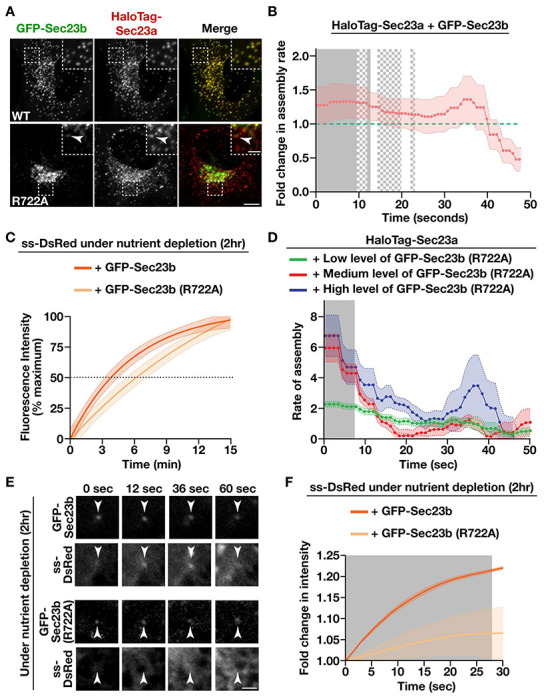
Increasing the rate of inner COPII coat assembly accelerates the rate of cargo egress from the ER during acute nutrient deprivation. (A) Representative images showing the relative distributions of two GFP-Sec23b isoforms as compared to natively expressed HaloTag-Sec23a following labeling with JFX650-HaloTag ligand. Arrowheads highlight a co-localization event. Bar, 5 μm; inset bar, 1 μm. (B) Quantification of the fold change in the assembly rate of HaloTag-Sec23a following 2 hour nutrient deprivation in the presence of exogenously expressed WT GFP-Sec23b and in its absence (represented by a dashed green line). Error bars represent mean +/− SEM (n=10 cells each; 3 biological replicates; more than 9,000 tracked particles each). Solid gray (p < 0.05) and checkered gray (p < 0.1) regions represent significant differences, calculated using multiple, unpaired *t* tests at each time point. (C) Confocal imaging of control cells expressing ss-DsRed in the presence of GFP-Sec23b or GFP-Sec23b (p.R722A) following 2 hours of nutrient deprivation was used to monitor its synchronous release from the ER. Based on fluorescence intensity, the percentage of ss-DsRed present within the perinuclear region relative to its maximal accumulation there was quantified over time. Error, displayed by lightly colored bands around smoothed tread lines, represents mean +/− SEM (n=20 cells each; 3 biological replicates each). (D) Quantification of the instantaneous rate of assembly of tracked HaloTag-Sec23a-positive structures exhibiting various levels of GFP-Sec23b (p.R722A) intensity. Error, as displayed by lightly colored bands around smoothed tread lines, represents mean +/− SEM (n=10 cells each; 3 biological replicates; more than 3000 tracked particles each). Solid gray (p < 0.05) regions represent significant differences compared to low levels of GFP-Sec23b (p.R722A) expression, calculated using multiple, unpaired *t* tests at each time point. (E) Representative timelapse images collected using TIRF microscopy showing the relative distributions of two GFP-Sec23b isoforms as compared to ss-DsRed following its release from the ER. Arrowheads highlight ER subdomains harboring GFP-Sec23b isoforms. Bar, 1 μm. (F) TIRF-based imaging of control cells expressing ss-DsRed in the presence of GFP-Sec23b or GFP-Sec23b (p.R722A) following 2 hours of nutrient deprivation was used to monitor its synchronous release from the ER. The fold change in ss-DsRed fluorescence intensity at individual ER subdomains marked by GFP-Sec23b isoforms relative to its maximal accumulation there was quantified over time. Error, displayed by lightly colored bands around smoothed tread lines, represents mean +/− SEM (n=30 cells each; 3 biological replicates; more than 4000 tracked events each). Solid gray (p < 0.05) regions represent significant differences, calculated using multiple, unpaired *t* tests at each time point.

To directly test the idea that the kinetics of Sec23a incorporation at ER subdomains functions as a rheostat that determines the speed of secretory cargo efflux, we developed an approach to artificially increase its assembly rate in cells deprived of nutrients for 2 hours. Specifically, based on previous work showing that overexpression alters the assembly rates of ESCRT complex subunits (68), we transduced cells natively expressing HaloTag-Sec23a with a virus encoding GFP-Sec23b. While fluorescence microscopy demonstrated that overexpressed GFP-Sec23b was incorporated into sites harboring endogenous HaloTag-Sec23a (Fig. 6a), live cell imaging failed to reveal significant changes in HaloTag-Sec23a lifetime, its frequency of formation, or its level of expression (Supplementary Fig. 8a–c). Nonetheless, the assembly rate of HaloTag-Sec23a was elevated significantly by overexpression of GFP-Sec23b, to a level similar to that observed following 24 hours of nutrient deprivation (Fig. 5e and 6b). Strikingly, the delay in ss-DsRed trafficking associated with short-term nutrient deprivation was resolved by solely overexpressing GFP-Sec23b (Fig. 3b and 6c; Supplementary Movie S8), strongly suggesting that the kinetics of Sec23 incorporation at ER subdomains controls the rate at which cargoes are able to exit the ER.

To determine whether the GAP activity of Sec23 is necessary for this effect, we overexpressed a mutant form of GFP-Sec23b, which harbors the R722A mutation that lacks GAP activity in vitro (49). Again, following stable transduction, we failed to identify any changes in the frequency of formation of HaloTag-Sec23a positive sites, but there was a modest increase in their lifetime, likely due to reduced GAP activity, which is necessary for transport intermediate maturation (Fig. 6a and Supplementary Fig. 8a–c). Interestingly, mutant GFP-Sec23b (p.R722A) expression acted similarly to wild-type GFP-Sec23b to increase the initial assembly rate of HaloTag-Sec23a and its lifetime at ER subdomains (Fig. 6d and Supplementary Fig. 8d), suggesting that Sec23 incorporation at ER subdomains does not rely on its GAP activity. Effects were concentration dependent, with higher levels of GFP-Sec23b (p.R722A) at ER subdomains resulting in faster assembly of natively-expressed HaloTag-Sec23a (Fig. 6d). However, Sec23 GAP activity was found to be essential for rescuing delays in cargo trafficking caused by short-term nutrient deprivation (Fig. 6c and Supplementary Movie S9). Based on total internal reflection fluorescence (TIRF) microscopy studies, cargoes failed to concentrate normally at ER subdomains harboring GFP-Sec23b (p.R722A) following their disaggregation (Fig. 6e, f), consistent with a requirement for tight control of Sar1 GTPase activity during transport intermediate formation, cargo loading, and anterograde trafficking at the ER/ERGIC interface (69).

## Discussion

Similar to gene transcription and protein translation, the nearly constitutive export of newly synthesized secretory proteins from the ER is essential for normal cellular homeostasis, growth, and development. Importantly, a variety of external stimuli have been shown to directly regulate each of these processes, enabling cells to react to changing environmental conditions (49–61). For example, in response to an immune challenge, B cells undergo differentiation to generate antibody-secreting plasma cells, which requires a dramatic change in gene expression patterns, expansion of the organelles that participate in membrane protein trafficking, and an increased flux through the COPII-mediated early secretory pathway (70). While several signaling networks that drive proliferation of ER membranes and/or modulate COPII subunit expression have been defined, mechanisms that directly control the rate of secretory protein packaging and export at individual sites of COPII transport intermediate biogenesis have remained poorly understood. To address this challenge, previous studies have relied mainly on the analysis of fixed cells grown under various conditions to describe impacts to the assembly of ER subdomains capable of secretory protein trafficking. In particular, the steady state levels of COPII components at these sites and the total number of subdomains found in cells have been used as a proxy for determining biosynthetic cargo export activity (61). While this work has been invaluable to identify regulators of COPII-mediated transport, the lifetime of COPII budding sites, their frequency of formation, and their rate of assembly have not been defined. Here, we reveal the dynamics of the COPII coat complexes under differing environmental conditions, showing that Sec23, which regulates GTP hydrolysis on Sar1, specifically plays a key role in coordinating the timing of transport intermediate biogenesis, even when the overall levels of other COPII components and regulators are reduced.

Under nutrient limiting conditions, several studies have identified key changes in Sec23 post-translational modification, which influence its ability to associate with other factors involved in COPII-mediated trafficking (51, 52, 57, 60). In particular, phosphorylation of Sec23B by ULK1 under conditions identical to those used in our nutrient deprivation studies, leads to its redistribution to ERGIC membranes, where it functions in the biogenesis of autophagosomes (52). Similarly, other forms of post-translational modification have been suggested to alter the ability of Sec23 to target to ER subdomains that produce COPII transport intermediates. Although relatively few studies have investigated impacts of prolonged nutrient deprivation, there is clear precedent for cells to adapt to environmental conditions, which may ultimately lead to further modifications on Sec23 that enable it to more efficiently support transport intermediate formation, even when its overall levels are dramatically lower (71). Further studies will be necessary to define the importance of potential sites of post-translational modification on Sec23 under varying nutrient availability conditions to validate this idea.

Based on our live cell imaging studies, the inner COPII coat persists at ER subdomains for nearly 60 seconds on average, similar to the 45-60 seconds necessary for clathrin-mediated endocytic pits to form and internalize from the surface of cells (72). In contrast, in vitro reconstitution studies suggest that the lifetime of assembled Sar1-Sec23-Sec24 complexes on membranes is substantially more transient, on average approximately 30 seconds (45). While we cannot exclude the possibility that the sites we identified in cells consist of multiple, simultaneous budding events, this is unlikely given their narrow range of individual lifetimes. Instead, the reconstituted inner COPII coat may disassemble prematurely in vitro due to the absence of regulatory factors, which influence COPII dynamics and GTP hydrolysis on Sar1. Our studies also revealed the relative longevity of some of these regulators at ER subdomains, indicating that Sec16a persists for a longer period of time as compared to COPII. These data support a model in which Sec16a functions to establish sites of COPII intermediate budding and facilitates the sequential recruitment of inner and outer COPII coat complexes. Consistent with this idea, Sec23 persists for a longer period of time at ER subdomains as compared to Sec31. Notably, the longest-lived components exhibited the fewest formation events, while those that appeared relatively transiently arose more often in cells, indicative of an inverse relationship between lifetime and frequency of assembly. This balance may help to ensure that at any given time, each component of the COPII machinery is found at nearly every ER budding site, which is commonly witnessed at steady state, despite the highly dynamic nature of COPII assembly.

To our surprise, the assembly kinetics of the COPII regulatory factor TFG were very distinct from its binding partner Sec23, exhibiting a significantly shorter lifetime at the ER/ERGIC interface. In previous work, we demonstrated that TFG functions to cluster COPII transport intermediates, while promoting their uncoating prior to fusion with ERGIC membranes (42, 43). Following short-term nutrient deprivation, when COPII-mediated trafficking was slowed, the rate of TFG assembly was paradoxically elevated, suggesting that TFG function may extend beyond its canonical roles in facilitating transport intermediate fusion with ERGIC membranes. One possibility is that increased TFG addition under nutrient limiting conditions interferes with COPII outer coat assembly, as TFG competes with Sec31 for binding to Sec23. This idea is consistent with the decreased frequency of Sec31 appearance following acute nutrient depletion. Whether TFG may function similarly under nutrient replete conditions to regulate COPII coat assembly will require additional study. Nevertheless, based on our work, we now have ideal platforms to resolve this and other questions focused on the regulation of COPII dynamics.

## Methods

### Cell culture and genome editing

CRISPR/Cas9-mediated genome editing of human hTERT-immortalized RPE1 cells (CRL-4000 from ATCC) grown in DMEM:F-12 media (nutrient replete) was conducted as described previously (68, 73). The following guide RNA (gRNA) sequences were used: 5’-CTCCACAATGACAACCTATT-3’ (targeting Sec23a), 5’-GACGGGACCGTCTGGGGCGG-3’ (targeting Sec16a), 5’-CTTTAACTTCATCCTGCTAA-3’ (targeting Sec31a), 5’-ATCCAACTGTCCGTTCATGG-3’ (targeting TFG), and 5’-TCTACCACAGAATAACACCC-3’ (targeting GRASP65). Clonal populations were isolated using fluorescence activated cell sorting (FACS), which was followed by live cell imaging to confirm the proper distribution of each fusion protein.

To measure proliferation rates, cells seeded at low density were treated with 2 μM Calcein AM (15 minutes every 24 hours) and imaged on an ImageXpress Micro 4 platform (Molecular Devices) using a 10x (0.3 NA) dry objective. MetaXpress software was used to count cells in an unbiased manner and proliferation rates were determined using GraphPad Prism software. To conduct nutrient deprivation studies, cells were washed and subsequently cultured in Earle’s Balanced Salt Solution (EBSS) for 2 or 24 hours.

### Fluorescence microscopy and image analysis

Confocal imaging studies were carried out on a Nikon ECLIPSE Ti2 spinning disk confocal microscope equipped with a 60x oil immersion objective (1.4 NA) and a Hamamatsu ORCA-Flash4.0 sCMOS camera. HaloTag fusion proteins were labeled using either JFX650- or JF635-HaloTag ligands provided by Dr. Luke Lavis. Cargo trafficking and immunostaining assays were performed as described previously (43, 64, 65, 73). All datasets acquired during imaging were Nyquist sampled. Validated antibodies directed against Sec24a (Santa Cruz Biotechnology; sc-169279), GM130 (BD Sciences; BD610823), ERGIC-53 (Santa Cruz Biotechnology; sc-66880), and PDIA3 (Proteintech; 15967-1-AP) were used. Super resolution imaging was performed on a Zeiss LSM 880 confocal system with Airyscan using a 63x (1.4 NA) oil immersion objective. Imaging datasets were acquired at Nyquist sampling using ZenBlue Z sampling recommendations, following by deconvolution and denoising using proprietary Zeiss algorithms. Light-sheet microscopy was conducted on a 3i Lattice LightSheet microscope equipped with a Hamamatsu ORCA-Flash4.0 V3 sCMOS camera. Beam alignment, dye alignment, and bead alignment were performed prior to imaging. All timelapse light-sheet studies were conducted with temporal resolutions of 3-4 seconds, 1024 x 1024 pixel resolution, and a step size of 297 nm in the Z plane. Images were deskewed and deconvolved using Slidebook software (3D frequency filter enabled, Gaussian smoothing at 0.6, and mirrored edge padding of 20%).

Fiji image processing software was used to quantify the accumulation of cargoes within the perinuclear region of cells (relative to their fluorescence maxima measured during imaging) and conduct linescan measurements. Cargo accumulation over time was fitted to an exponential plateau using GraphPad Prism software. Imaris software was used to measure intensities, volumes, and the density of structures within cells. To quantify the dynamics of HaloTag fusion proteins at ER subdomains, light-sheet imaging datasets were time normalized, and only structures that assembled and disassembled during each imaging session were analyzed. Assembly curves were generated based on intensity measurements and smoothed using fine Locally Weighted Scatterplot Smoothing (LOWESS). Derivatives were calculated using GraphPad Prism and used for statistical analysis. Unless otherwise noted, images shown are representative of 3 biological replicates (minimally 10 cells each).

### Biochemistry and molecular biology

Immunoblotting studies were conducted as described previously (43). The following validated antibodies were used: Sec31a (BD Sciences; 612351), Sec23a (Thermo Scientific; PA5-28984), TFG (Novus Biologicals; NBP2-62212), β-actin (Sigma; A1978), GAPDH (Proteintech; 60004-1), and GRP78/BIP (Proteintech; 11587-1-AP).

RNA extraction was performed using TRIzol (Invitrogen), followed by sequential precipitations using ethanol and lithium chloride. Production of cDNA was performed using a Superscript III First Strand RT-PCR kit (Invitrogen), and RT-qPCR experiments were performed using a CFX384 Touch Real-time PCR detection system (Bio-Rad) and Applied Biosystems Power SYBR Green PCR Master Mix (Thermo Scientific). The following primers were used: sXBP1 F: CCCTCCAGAACATCTCCCCAT; sXBP1 R: ACATGACTGGGTCCAAGTTGT; GAPDH F: AGCCACATCGCTCAGACAC; GAPDH R: GCCCAATACGACCAAATCC.

## Supplementary Material

1

## Data Availability

All reagents generated in this study are available from the corresponding author with a Material Transfer Agreement. All data supporting the key findings of this study are available within the article and its Supplementary Information files.
