# Supplementary material for: Nutrient deprivation alters the rate of COPII coat assembly to tune secretory protein transport
Source: Res Sq. 2023 Mar 17:rs.3.rs-2652351. Preprint. [Version 1] doi: 10.21203/rs.3.rs-2652351/v1 (PMC10055522; doi:10.21203/rs.3.rs-2652351/v1)
Supplement: 1 [file NIHPPrs2652351v1-supplement-1.pdf]

Supplementary Figure Legends

Figure S1. Appending HaloTag onto endogenous COPII subunits fails to impact their function. (A) Cartoons depicting the strategy used to created HaloTag fusions with native Sec16a, Sec23a, Sec31a, and TFG. (B) Representative immunoblot (n=3) of extracts generated from control and clonal CRISPR/Cas9 edited cell lines using antibodies directed against Sec31a (top) and β-actin (bottom). (C) PCR was used to analyze genomic DNA from control cells and clonal cells expressing a HaloTag fusion to Sec16a. An asterisk highlights a nonspecific band, based on sequence analysis. (D) The proliferation rates of control cells and genome edited cells expressing HaloTag fusion proteins were determined using widefield imaging. Error bars represent the mean +/- SEM (at least 3 biological replicates each). ***, $p < 0.001$ as calculated using an ANOVA and Tukey post hoc test. (E) Based on confocal microscopy, the total number of structures decorated by each HaloTag fusion protein following labeling with JFX650-HaloTag ligand was calculated throughout the volume of cells (n=20 cells each; 3 biological replicates each). No statistically significant differences were found, as calculated using an ANOVA and Tukey post hoc test.

Figure S2. Genome edited cell lines exhibit normal organization of the early secretory pathway. (A) Representative confocal images of cell lines natively expressing HaloTag fusion proteins co-stained with antibodies directed against endogenous GM130 (red) and ERGIC-53 (green). Bar, 10 μm. (B) Based on confocal microscopy, the total number of structures decorated by ERGIC-53 (top) and the total cellular volume labeled by GM130 (bottom) was calculated within cells expressing HaloTag fusions proteins (n=15 cells each; 3 biological replicates each). No statistically significant differences were found, as calculated using an ANOVA and Tukey post hoc test. (C) Representative super resolution images of fixed cells

expressing HaloTag fusion proteins labeled with the JFX650-HaloTag ligand and co-stained using antibodies directed against PDIA3. Bar, 1 μm.

Figure S3. Genome edited cell lines exhibit normal trafficking of secretory cargoes. (A and B) Representative confocal images of control cells and cell lines natively expressing HaloTag fusion proteins co-expressing ss-DsRed (A) or ManII-SBP-GFP (B) at various timepoints following their release from the ER. Zoomed images of the perinuclear region are shown. Bar, 5 μm.

Figure S4. Dynamics of HaloTag fusion proteins at ER subdomains. (A-D) Fluorescence intensity of HaloTag-Sec16a (A), HaloTag-Sec23a (B), HaloTag-Sec31a (C), and HaloTag-TFG (D) positive structures tracked over time using light-sheet imaging following labeling with JFX650-HaloTag ligand. Error bars represent mean +/- SEM (n=10 cells each; more than 3000 tracked structures each; 3 biological replicates).

Figure S5. Acute and long-term nutrient deprivation differentially influence the rate of secretory cargo trafficking. (A and B) Confocal imaging of control cells expressing either ss-DsRed (A) or ManII-SBP-GFP (B) was used to monitor their synchronous release from the ER in the presence of absence of nutrients. Based on fluorescence intensity, the percentage of each cargo present within the perinuclear region relative to its maximal accumulation there was quantified over time and fitted to an exponential with the dotted line indicating half-maximal intensity. Error, as displayed by lightly colored bands, represent mean +/- SEM (n=30 cells each; 3 biological replicates each). (C) Quantification of the half-time to perinuclear accumulation of ManII-SBP-GFP under various nutrient availability conditions. Error bars represent mean +/- SEM (n=30 cells each; 3 biological replicates each). **, $p < 0.01$ and *, p <

0.05, calculated using a one-way ANOVA and Tukey post hoc test. (D) Representative confocal images of control cells (perinuclear region) in the presence and absence of nutrients expressing a releasable form of HaloTag-L1CAM (labeled with JFX650-HaloTag ligand) before and after its release from the ER. Bar, 2 μm. (E) Based on fluorescence measurements, the fold change in HaloTag-L1CAM intensity (following labeling with JFX650-HaloTag ligand) within the perinuclear region was quantified over time in the presence and absence of nutrients. Error bars represent mean +/- SEM (n=15 cells each; 3 biological replicates each). ****, $p < 0.0001$; **, $p < 0.01$ and *, $p < 0.05$, calculated using a one-way ANOVA and Tukey post hoc test. (F) Representative super resolution images of clonal CRISPR/Cas9-edited cells expressing GRASP65-HaloTag in the presence and absence of nutrients following labeling with JFX650-HaloTag ligand. Bar, 5 μm.

Figure S6. Nutrient deprivation reduces expression of COPII subunits. (A) Levels of sXBP1 mRNA in nutrient deprived cells were determined relative to control cells using quantitative PCR. Error bars represent mean +/- SEM, based on 3 biological replicates. No statistically significant differences were found, as calculated using an unpaired *t* test. (B) Levels of GRP78 in nutrient deprived cells were determined relative to control cells using quantitative immunoblotting. Error bars represent mean +/- SEM, based on 3 biological replicates. No statistically significant differences were found, as calculated using an unpaired *t* test. (C) Representative immunoblots (n=3 each) of extracts generated from control cells in the presence and absence of nutrients using antibodies directed against Sec23a (top), TFG (middle) and β-actin (bottom). (D) Quantification of immunoblots shown in panel C. Error bars represent mean +/- SEM, based on 3 biological replicates. ****, $p < 0.0001$, calculated using a one-way ANOVA and Tukey post hoc test. (E) Based on quantitative fluorescence measurements, the intensity of each HaloTag fusion protein was compared in the presence and absence of nutrients. Error

bars represent mean +/- SEM (n=15 cells each; 3 biological replicates each). ***, $p < 0.001$ and *, $p < 0.05$, calculated using a one-way ANOVA and Tukey post hoc test.

Figure S7. Long-term nutrient deprivation reduces the number of COPII-positive structures in cells, but elevates their intensities. (A) Representative confocal images of cell lines natively expressing HaloTag fusion proteins after labeling with JFX650-HaloTag ligand in the presence and absence of nutrients are shown. Bar, 5 μm. (B-D) Based on confocal microscopy, the total number of structures decorated by each HaloTag fusion protein (B), their intensities (C) and their volumes (D) were calculated. Error bars represent mean +/- SEM (n=30 cells each; 3 biological replicates each). ***$p < 0.001$; **$p < 0.01$ and *$p < 0.05$, calculated using a one-way ANOVA and Tukey post hoc test.

Figure S8. Overexpression of GFP-Sec23b does not influence the distribution of natively expressed HaloTag-Sec23a. (A) Quantification of the average duration of HaloTag-Sec23a at ER subdomains in the presence or absence of overexpressed GFP-Sec23b isoforms following acute nutrient deprivation. Error bars represent mean +/- SEM (n=20 cells each; 3 biological replicates). **, $p < 0.01$, as calculated using a one-way ANOVA and Tukey post hoc test. (B) Quantification of the number of HaloTag-Sec23a positive structures that assemble each minute in the presence or absence of overexpressed GFP-Sec23b isoforms following acute nutrient deprivation. Error bars represent mean +/- SEM (n=20 cells each; 3 biological replicates). No statistically significant differences were found, as calculated using an unpaired *t* test. (C) The total cellular intensity of HaloTag-Sec23a following labeling with JFX650-HaloTag ligand was determined in the presence or absence of overexpressed GFP-Sec23b. (D) Quantification of the average duration of HaloTag-Sec23a at ER subdomains in the presence or absence of varying levels of GFP-Sec23b (p.R722A) following acute nutrient deprivation. Error bars represent mean

+/- SEM (n=20 cells each; 3 biological replicates). ***, $p < 0.001$, as calculated using a one-way ANOVA and Tukey post hoc test.

Supplementary Movie Legends

Movies S1-S4. Dynamics of HaloTag-Sec16a (Movie S1), HaloTag-Sec23a (Movie S2), HaloTag-Sec31a (Movie S3), and HaloTag-TFG (Movies S4) as determined using lattice light-sheet imaging (n=10 cells each; 3 biological replicates each). Bar, 10 μm. Playback rate, 180x.

Movies S5-S7. Accumulation of ss-DsRed in the perinuclear region of control cells following its release in the presence (Movie S5) and absence of nutrients for 2 hours (Movie S6) or 24 hours (Movie S7). n=20 cells each; 3 biological replicates each. Bar, 10 μm. Playback rate, 180x.

Movies S8-S9. Accumulation of ss-DsRed in the perinuclear region of control cells following its release in the acute absence of nutrients following overexpression of either GFP-Sec23b (Movie S8) or GFP-Sec23b p.R722A (Movie S9). n=20 cells each; 3 biological replicates each. Bar, Bar, 10 μm. Playback rate, 180x.

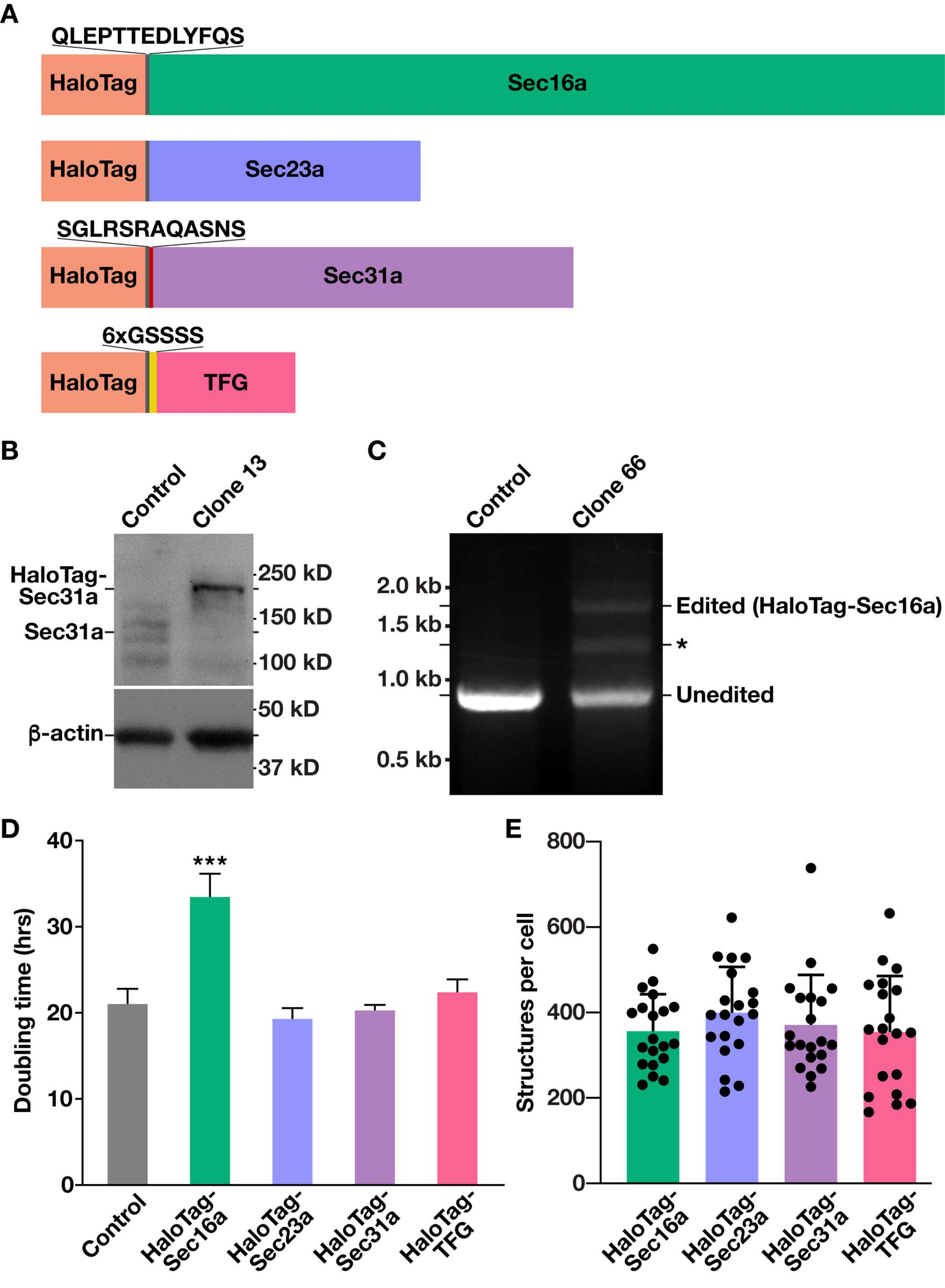

A
QLEPTTEDLYFQS
HaloTag
Sec16a
HaloTag
Sec23a
SGLRSRAQASNS
HaloTag
Sec31a
6xGSSSS
HaloTag
TFG
B
Control
Clone 13
HaloTag-Sec31a
Sec31a
250 kD
150 kD
100 kD
50 kD
β-actin
37 kD
C
Control
Clone 66
2.0 kb
1.5 kb
1.0 kb
0.5 kb
Edited (HaloTag-Sec16a)
*
Unedited
D
Doubling time (hrs)
40
30
20
10
0
***
Control
HaloTag-Sec16a
HaloTag-Sec23a
HaloTag-Sec31a
HaloTag-TFG
E
Structures per cell
800
600
400
200
0
HaloTag-Sec16a
HaloTag-Sec23a
HaloTag-Sec31a
HaloTag-TFG

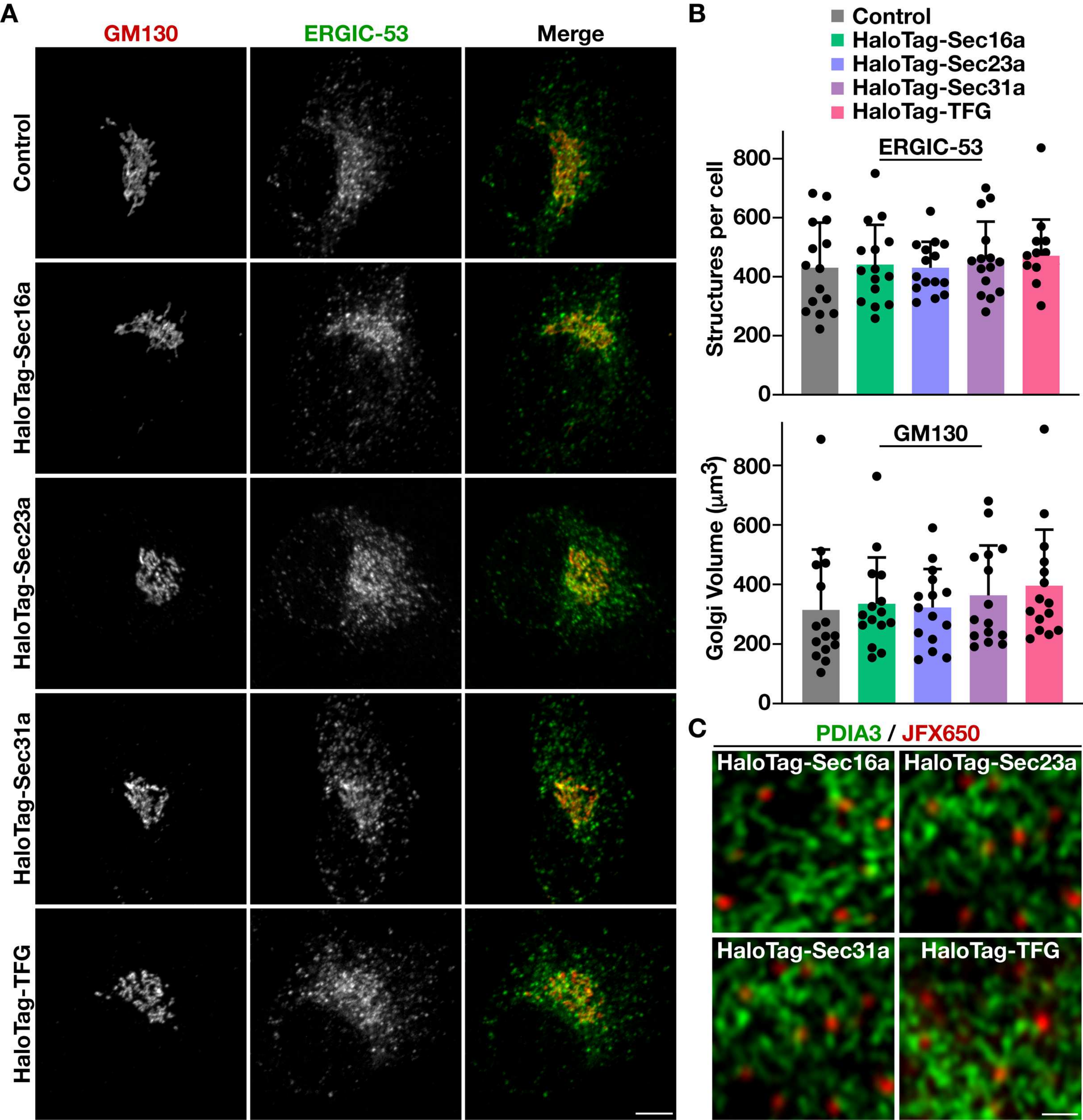

A
GM130
ERGIC-53
Merge
Control
HaloTag-Sec16a
HaloTag-Sec23a
HaloTag-Sec31a
HaloTag-TFG
B
Control
HaloTag-Sec16a
HaloTag-Sec23a
HaloTag-Sec31a
HaloTag-TFG
ERGIC-53
Structures per cell
0
200
400
600
800
GM130
Golgi Volume (μm3)
0
200
400
600
800
C
PDIA3 / JFX650
HaloTag-Sec16a
HaloTag-Sec23a
HaloTag-Sec31a
HaloTag-TFG

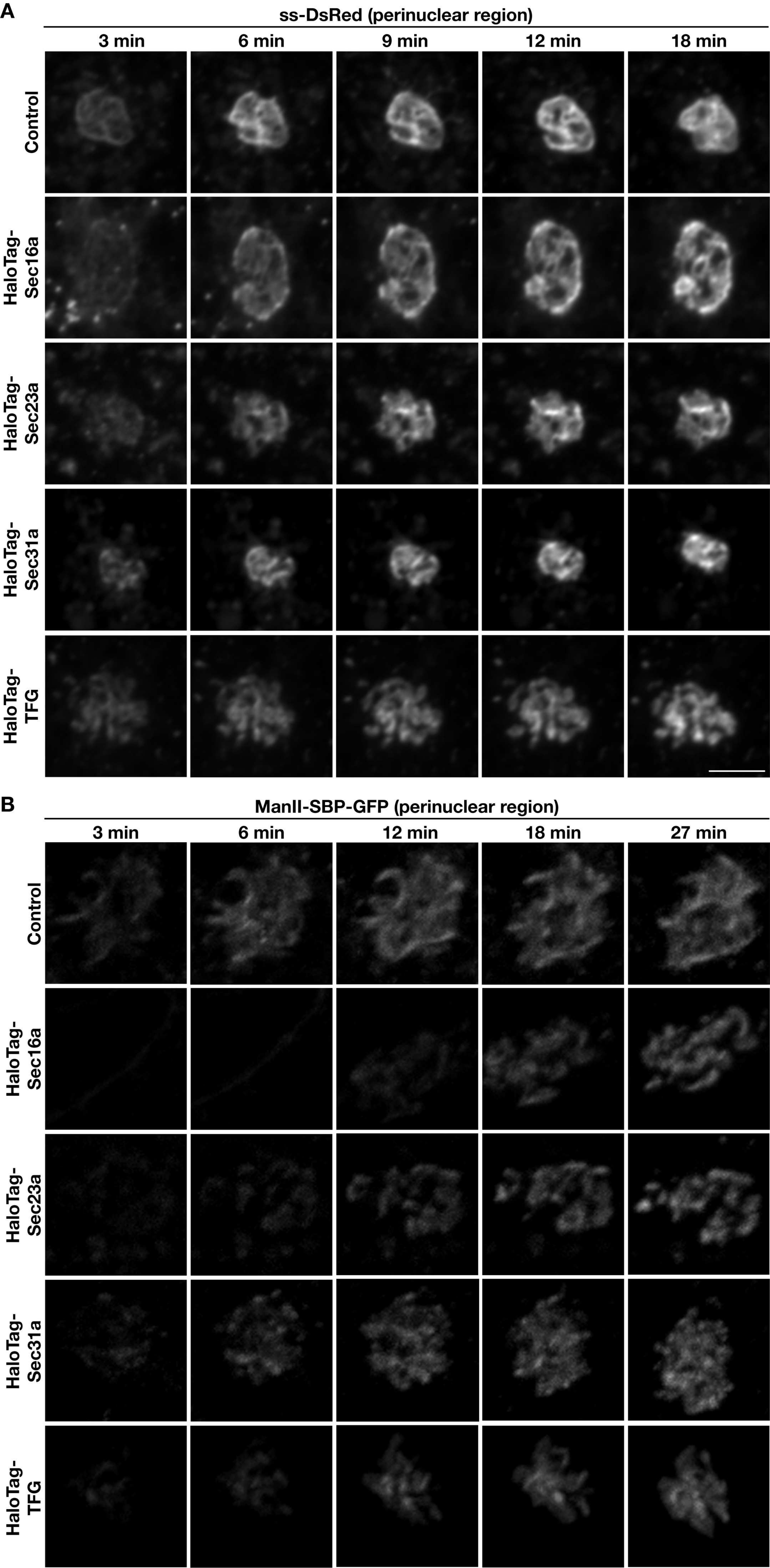
A
ss-DsRed (perinuclear region)
3 min
6 min
9 min
12 min
18 min
Control
HaloTag-Sec16a
HaloTag-Sec23a
HaloTag-Sec31a
HaloTag-TFG
B
ManII-SBP-GFP (perinuclear region)
3 min
6 min
12 min
18 min
27 min
Control
HaloTag-Sec16a
HaloTag-Sec23a
HaloTag-Sec31a
HaloTag-TFG

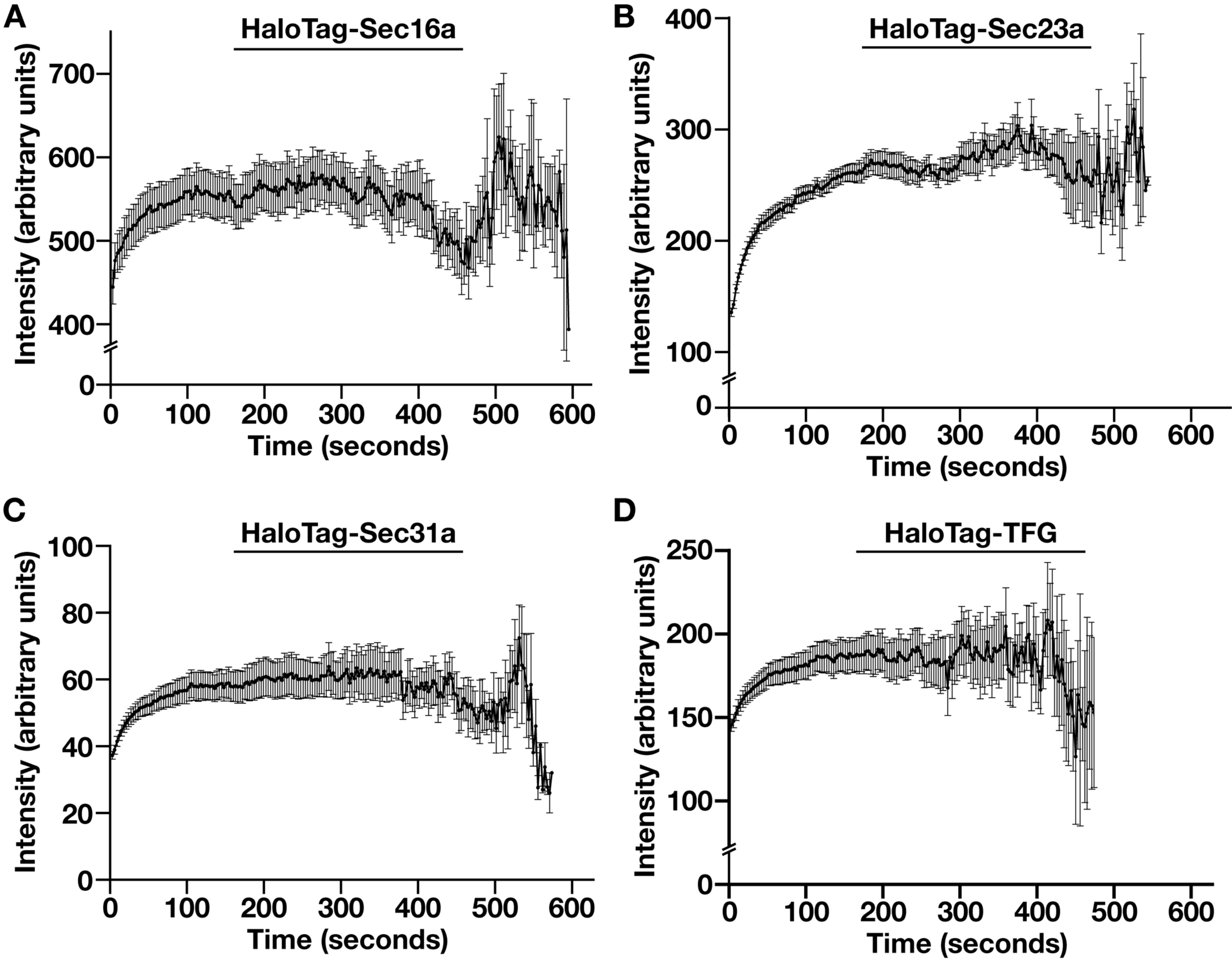
A
HaloTag-Sec16a
Intensity (arbitrary units)
700
600
500
400
0
0
100
200
300
400
500
600
Time (seconds)
B
HaloTag-Sec23a
400
300
200
100
0
Intensity (arbitrary units)
0
100
200
300
400
500
600
Time (seconds)
C
HaloTag-Sec31a
100
80
60
40
20
0
Intensity (arbitrary units)
0
100
200
300
400
500
600
Time (seconds)
D
HaloTag-TFG
250
200
150
100
0
Intensity (arbitrary units)
0
100
200
300
400
500
600
Time (seconds)

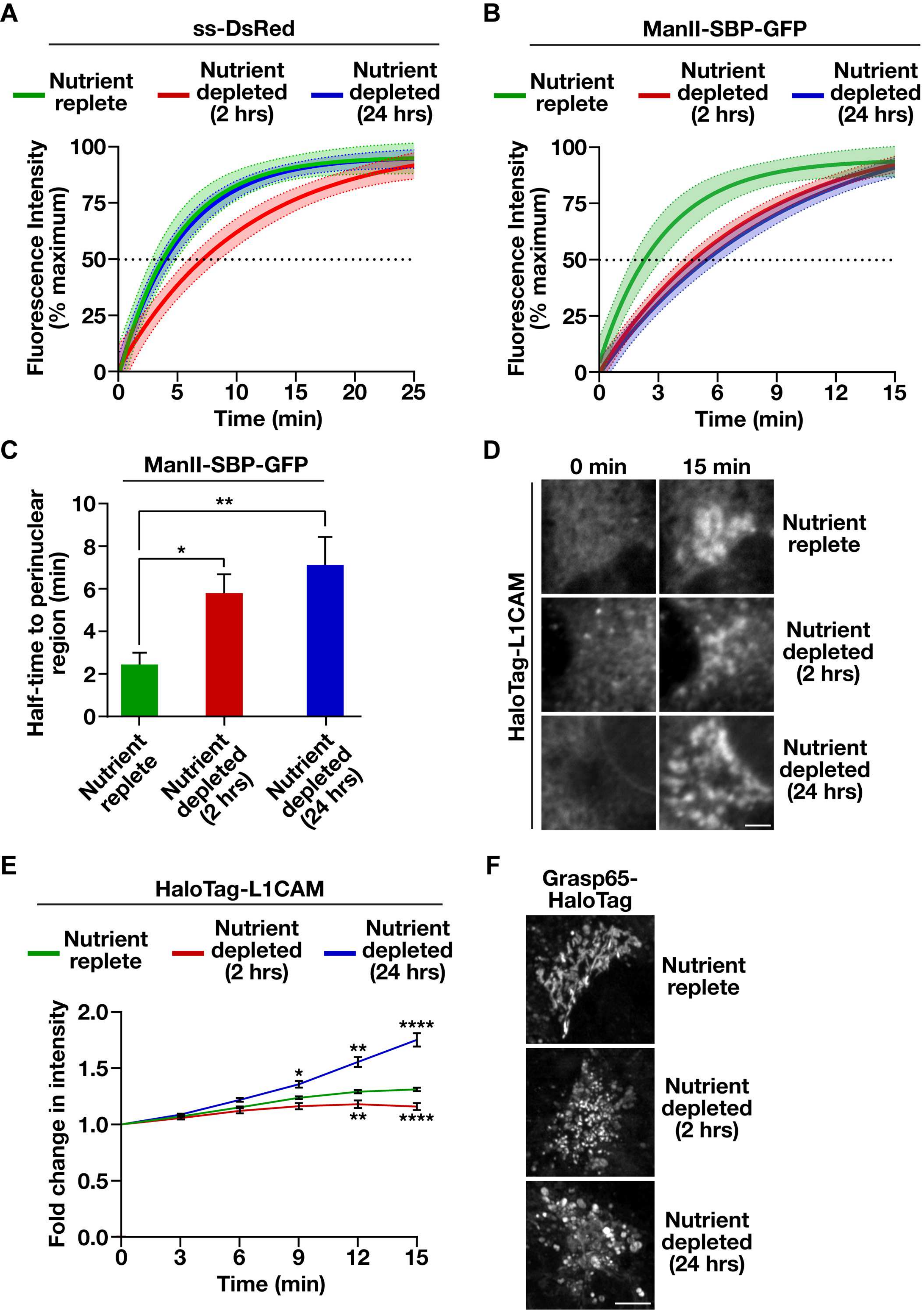
A
ss-DsRed
Nutrient replete
Nutrient depleted (2 hrs)
Nutrient depleted (24 hrs)
Fluorescence Intensity (% maximum)
Time (min)
B
ManII-SBP-GFP
Nutrient replete
Nutrient depleted (2 hrs)
Nutrient depleted (24 hrs)
Fluorescence Intensity (% maximum)
Time (min)
C
ManII-SBP-GFP
Half-time to perinuclear region (min)
Nutrient replete
Nutrient depleted (2 hrs)
Nutrient depleted (24 hrs)
D
0 min
15 min
HaloTag-L1CAM
Nutrient replete
Nutrient depleted (2 hrs)
Nutrient depleted (24 hrs)
E
HaloTag-L1CAM
Nutrient replete
Nutrient depleted (2 hrs)
Nutrient depleted (24 hrs)
Fold change in intensity
Time (min)
F
Grasp65-HaloTag
Nutrient replete
Nutrient depleted (2 hrs)
Nutrient depleted (24 hrs)

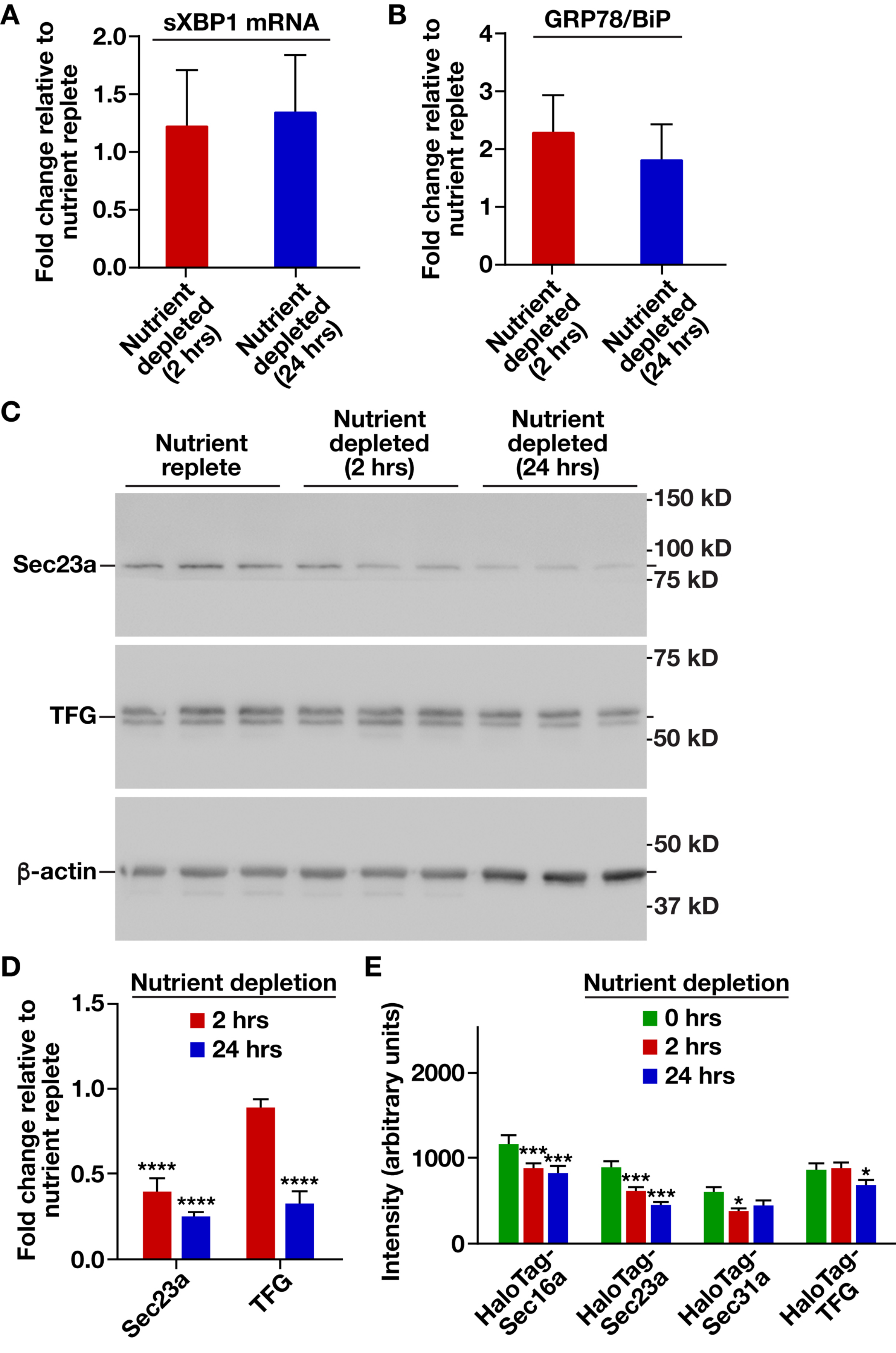
A
sXBP1 mRNA
Fold change relative to nutrient replete
2.0
1.5
1.0
0.5
0.0
Nutrient depleted (2 hrs)
Nutrient depleted (24 hrs)
B
GRP78/BiP
Fold change relative to nutrient replete
4
3
2
1
0
Nutrient depleted (2 hrs)
Nutrient depleted (24 hrs)
C
Nutrient replete
Nutrient depleted (2 hrs)
Nutrient depleted (24 hrs)
150 kD
100 kD
Sec23a
75 kD
75 kD
TFG
50 kD
50 kD
β-actin
37 kD
D
Nutrient depletion
2 hrs
24 hrs
Fold change relative to nutrient replete
1.5
1.0
0.5
0.0
****
****
****
Sec23a
TFG
E
Nutrient depletion
0 hrs
2 hrs
24 hrs
Intensity (arbitrary units)
2000
1000
0
***
***
***
***
*
*
HaloTag-Sec16a
HaloTag-Sec23a
HaloTag-Sec31a
HaloTag-TFG

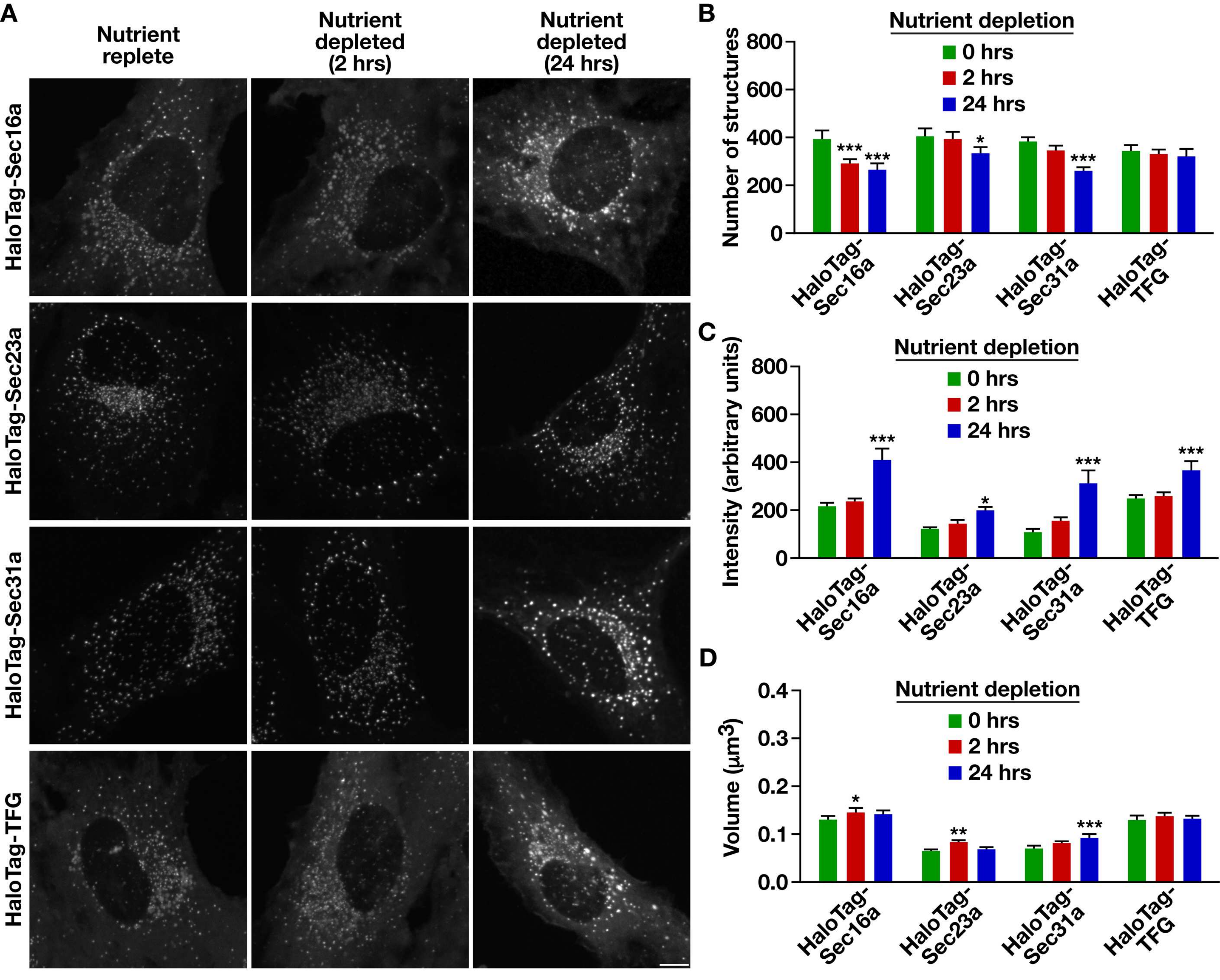
A
Nutrient replete
Nutrient depleted (2 hrs)
Nutrient depleted (24 hrs)
HaloTag-Sec16a
HaloTag-Sec23a
HaloTag-Sec31a
HaloTag-TFG
B
Nutrient depletion
0 hrs
2 hrs
24 hrs
Number of structures
800
600
400
200
0
***
***
*
***
HaloTag-Sec16a
HaloTag-Sec23a
HaloTag-Sec31a
HaloTag-TFG
C
Nutrient depletion
0 hrs
2 hrs
24 hrs
Intensity (arbitrary units)
800
600
400
200
0
***
*
***
***
HaloTag-Sec16a
HaloTag-Sec23a
HaloTag-Sec31a
HaloTag-TFG
D
Nutrient depletion
0 hrs
2 hrs
24 hrs
Volume (µm3)
0.4
0.3
0.2
0.1
0.0
*
**
***
HaloTag-Sec16a
HaloTag-Sec23a
HaloTag-Sec31a
HaloTag-TFG

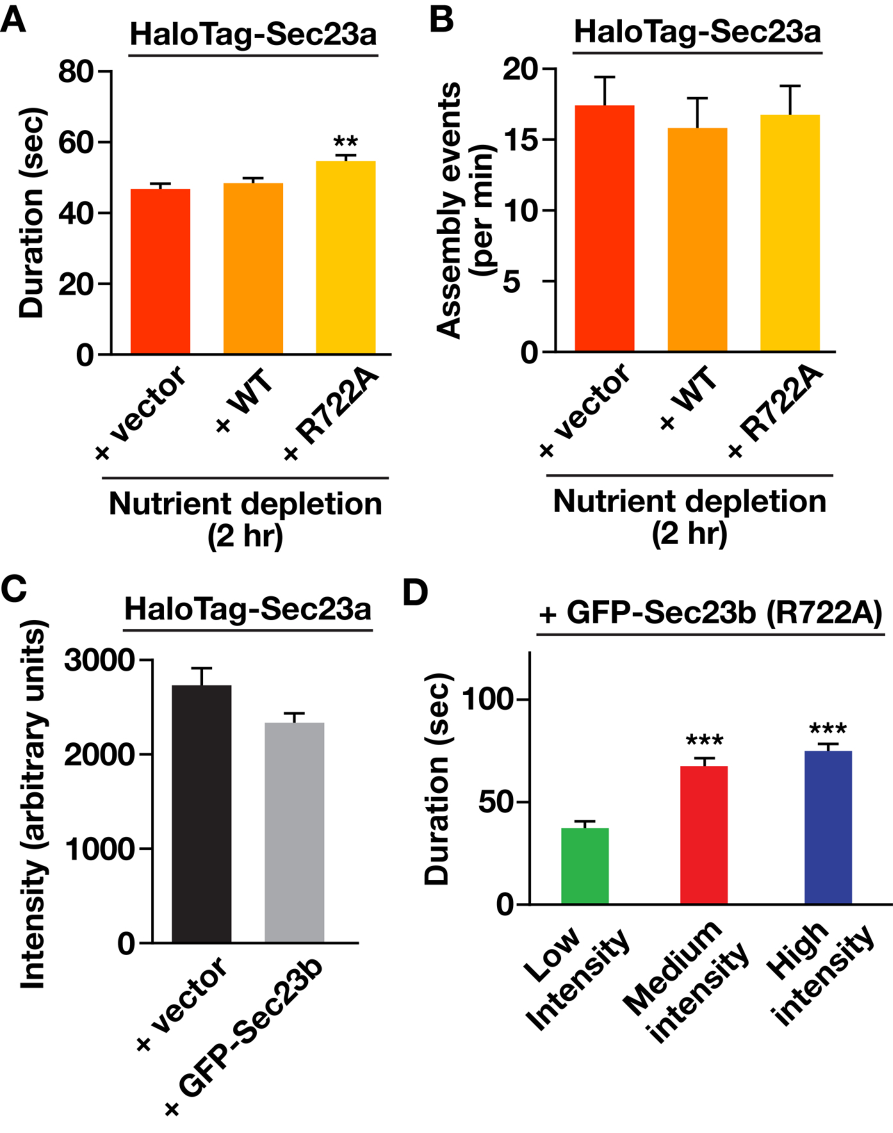

A
HaloTag-Sec23a
Duration (sec)
80
60
40
20
0
**
+ vector
+ WT
+ R722A
Nutrient depletion (2 hr)
B
HaloTag-Sec23a
Assembly events (per min)
20
15
10
5
0
+ vector
+ WT
+ R722A
Nutrient depletion (2 hr)
C
HaloTag-Sec23a
Intensity (arbitrary units)
3000
2000
1000
0
+ vector
+ GFP-Sec23b
D
+ GFP-Sec23b (R722A)
Duration (sec)
100
50
0
***
***
Low Intensity
Medium intensity
High intensity
